# Evaluation of Using Behavioural Changes to Assess Post-Operative Pain in the Guinea Pig (*Cavia porcellus*)

**DOI:** 10.1371/journal.pone.0161941

**Published:** 2016-09-01

**Authors:** Yvette Ellen, Paul Flecknell, Matt Leach

**Affiliations:** 1 QIMR Berghofer Medical Research Institute, Queensland, Australia; 2 Institute of Neuroscience, Newcastle University, Newcastle, United Kingdom; 3 School of Agriculture, Food & Rural Development, Newcastle University, Newcastle, United Kingdom; Harvard University Faculty of Arts and Sciences, UNITED STATES

## Abstract

To manage pain effectively in people and animals, it is essential to recognise when pain is present and to assess its intensity. Currently there is very little information regarding the signs of post-surgical pain or its management in guinea pigs. Studies from other rodent species indicate that behaviour-based scoring systems can be used successfully to detect pain and evaluate analgesic efficacy. This preliminary study aimed to establish whether behaviour-based scoring systems could be developed to assess post-surgical pain in guinea pigs. This prospective, randomised, placebo-controlled study used 16 guinea pigs, and evaluated changes in behaviour following either anaesthesia alone or anaesthesia and orchiectomy. Behaviour was assessed using a combination of manual and automated scoring of remotely obtained video footage. A small number of behaviours were identified that appeared to have high specificity for pain caused by orchiectomy. However, the behaviours were displayed infrequently. The most common was a change in posture from standing to recumbency, sometimes with one hind leg extended either to the side or behind the body. A composite behaviour score incorporating these abnormal behaviours differentiated between the effects of surgery and anaesthesia alone (p<0.0001), and between animals that received analgesia post-operatively compared to an untreated group (p<0.0001). Although behavioural changes occurred in these guinea pigs after orchiectomy, the changes were relatively subtle and the individual specific pain-related behaviours occurred infrequently. However, it may prove possible to develop a behaviour-based scoring system for routine use in this species using a combination of pain-related behaviours.

## Introduction

The guinea pig (*Cavia porcellus*) is a species that is used both in biomedical research and is often kept as a companion animal. For example, in the UK in 2014 over approximately 27,000 guinea pigs were used in regulated research [1] and 700,000 were kept as pets [2]. Surgical procedures are likely to cause pain in guinea pigs, as in other mammalian species, and analgesics should be administered to alleviate this pain. In order to administer effective pain relief, it is important to be able to evaluate effectively and compare the presence and intensity of pain, both before and after administration of analgesic therapy. Although guinea pigs often undergo routine surgeries such as ovariohysterectomy and orchiectomy in veterinary clinical practice, and a range of different surgical procedures as part of biomedical research projects, there are few objective, evidence-based recommendations for pain recognition and no evidence-based analgesic regimens in this species. According to the Committee on Recognition and Alleviation of Pain in Laboratory Animals, “analgesia in guinea pigs remains a purely empirical exercise based on anecdote, experience and best practice” [3]. In comparison, pain related behavioural and changes in facial expressions have been recorded in response to surgical procedures in a number of laboratory animal species such as mice [4,5,6] rats [7,8,9,10] and rabbits [11,12]. Post-operative weight loss and decreased water consumption has also been recorded in the rat after laparotomy [13] and weight loss and decreased food intake in mice after laparotomy [14]. These findings, especially in combination, provide a useful framework for the evaluation of existing and new analgesic regimens in these species with the potential for the refinement of post-operative pain management.

In contrast, very limited data is available in the guinea pig. An unpublished doctoral thesis has reported a higher frequency of food and water intake and greater weight gain post-surgery in guinea pigs medicated with meloxicam (a non-steroidal anti-inflammatory drug) compared to negative controls [15]. No significant differences in physiological parameters such as heart and respiratory rate were found or of pain intensity assessed using Visual Analogue Scores and Numerical Rating Scores between treated and control guinea pigs [15]. A more detailed evaluation of behaviours before and after surgery reported changes in specific activities (abdominal contraction, back arching, twitching and weight shifting), but no assessment of the effects of analgesics was undertaken [16].

In order to refine the post-surgical care of this species in both the biomedical and veterinary setting, it is necessary to develop more specific methods of detecting and quantifying pain as we have done with other species [4, 7] since the non-specific measures that can be taken from generic scoring systems such as that described by Morton and Griffiths [17] are likely to be unsatisfactory for determination of analgesic efficacy. These schemes generally select a range of clinical signs, and assign a numerical score using predefined criteria for changes from normality. The total score is considered to provide an indication of the welfare state of the animal. The changes identified by such scoring systems provide a useful assessment of the clinical condition of the animal, but do not confirm whether these changes are due to pain, or to other underlying conditions (eg disease states).

This study evaluated the behavioural changes following orchiectomy surgery in the guinea pig with the purpose of developing a pain scoring system for this species. For ethical reasons, to limit unalleviated post-surgical pain in this preliminary study, rescue analgesia using buprenorphine (0.05mg/kg sc) was provided to the negative control (no-analgesia) group after the first post-surgery behaviour recording. In addition, both groups were treated with meloxicam 24h following surgery. We hypothesised that pain would result in changes in normal behaviour and that similar abnormal behaviours to those seen in the rat [7,8,9,10], mouse [4,5,6] and rabbit [11,12] would be observed. If these abnormal behaviours were observed only following surgery (ie, not after anaesthesia alone), and showed a reduction in their frequency or duration in animals that had received analgesics in comparison to a placebo control group, this would provide evidence that they were pain-related. Similarly, if the changes in normal behaviour occurred only following surgery, and were closer to pre-surgery frequencies or durations after use of analgesics, this would also indicate the changes were related to pain. In order to maximise the likelihood of detecting pain related behavioural changes, detailed manual recording of all behaviours noted was conducted, as well as an automated analysis of activity.

## Materials and Methods

### Ethical statement

All procedures were conducted in accordance with the United Kingdom’s 1986 Animals (Scientific Procedures) Act, (Project License PPL 60/4431) and approved by the Newcastle University Animal Welfare and Ethical Review Board. All guinea pigs were rehomed after the study was completed and rehoming was authorised by both the Project licence and Local Ethical Review Committee. This study employed a strict ‘rescue’ analgesia policy. Buprenorphine (0.05mg/kg subcutaneously (sc)) was administered to all animals in the placebo (no analgesia group) after the first 1h observation period following surgery, irrespective of whether they appeared to be experiencing pain and an any animal in the analgesic group that was deemed to be in greater then mild pain after the initial 1h observation period (assessed by an independent veterinarian). The number of animals used in this preliminary study were estimated based on the assumption that the differences of frequency of abnormal behaviours, and the variance within treatment groups would be similar to those previously reported in rats, hence we calculated that 8 animals per group would provide 80% power and detection of a significant treatment effect at p<0.05.

### Animals and husbandry

Young (3–6 week) adult male Dunkin-Hartley guinea pigs (n = 16) were included in the study, supplied by Harlan, Loughborough, UK. They weighed between 270-490g at the commencement of the study. Guinea pigs were individually housed in floor pens, with wire mesh sides giving visual access to other guinea pigs. Pens were constructed in house with dimensions of 90x60cm. Dark coloured bedding material Cellu-Dri Soft (Shepherd Speciality Papers, Richland, Michigan, USA) was used in the pens to create contrast between the guinea pig and the background to assist automated video tracking. Guinea pigs were given an acclimation period of one week during which time they were habituated to handling, filming pens and video cameras and the movement of the camera panning equipment. Filming pens had similar construction and were the same size as holding pens but had had two Perspex sides to allow unobstructed views of the animals during filming. Guinea pigs were housed with a cardboard box shelter but habituated to its removal for 1 hour twice daily as no shelter was present in the pen during filming. Hay was provided daily as additional enrichment, and the food hopper was located within the pen. In the home pen each guinea pig also had a chew block and received fruit (pear, orange, apples).

The animals were free from common pathogens in accordance with FELASA health monitoring recommendations [18].

### Treatment groups

Random number generation was used to assign the guinea pigs to one of two treatment groups (Excel, Microsoft). Group 1 (negative control, n = 8) received saline sc one hour prior to surgery and saline infiltration of the surgical site at the time of surgery. Group 1 received rescue analgesia with buprenorphine (Vetergesic, Reckitt Benckiser Healthcare, Hull, UK) 0.05mg/kg sc after the first post-surgery behaviour recording (1h post-op). Group 2 (analgesic treated, n = 8) received meloxicam (Metacam, Boehringer Ingelheim, Berkshire, UK) 0.2mg/kg sc (diluted 1 in 10 with saline) one hour prior to surgery and infiltration with 1mg/kg bupivacaine hydrochloride (Marcaine Polyamp Steripack 0.25% AstraZeneca Ltd, Manchester, UK) and 4mg/kg lidocaine 1% hydrochloride (Hameln Pharmaceuticals Ltd, Gloucester, UK) inter-operatively. The local anaesthetics were mixed and diluted 1:1:2 (bupivacaine: lidocaine: saline) prior to use. Both groups received meloxicam 0.2mg/kg sc on the day after surgery. Table 1 summarises the procedures for each treatment group. The study was split into two phases; Phase 1 (placebo controlled phase) included baseline, pre and post anaesthesia and pre and post surgery), Phase 2 (Day post-surgery, pre and post meloxicam administration).

**Table 1:** Summary of procedures for the two treatment groups.

	Time-point	Group 1 Negative Control	Group 2 Analgesia Treated
**Phase 1**	Baseline	Filmed for 50 min (time matched with 1h post surgery/ anaesthesia time point)	Filmed for 50 min (time matched with 1h post surgery/ anaesthesia time point)
	Anaesthesia (6 days after baseline)	Weighed Injected with saline 1 hour before anaesthesia 20 min anaesthesia Fur clipped Filmed 1 hour post anaesthesia for 50 min Filmed 5 hours post anaesthesia for 50 min	Weighed Injected with meloxicam 1 hour before anaesthesia 20min anaesthesia Fur clipped Filmed 1 hour post anaesthesia for 50 min Filmed 5 hours post anaesthesia for 50 min
	Day post anaesthesia	Weighed	Weighed
	Surgery (7–15 days after anaesthesia)	Weighed Injected with saline 1 hour before surgery Surgery with saline infiltration Filmed 1 hour post surgery for 50 min Rescue buprenorphine immediately after filming Filmed 5 hours post surgery for 50 min	Weighed Injected with meloxicam 1 hour before surgery Surgery with local anaesthetic infiltration Filmed 1 hour post surgery for 50 min Saline injection immediately after filming Filmed 5 hours post surgery for 50min
**Phase 2**	Day post-surgery	Weighed Filmed for 50 min (time matched with 1h post time point) Meloxicam 1 hour before next filming Filmed for 50 min (time matched with 5h post time point)	Weighed Filmed (for 50 min (time matched with 1h post time point) Meloxicam 1 hour before next filming Filmed for 50 min (time matched with 5h post time point)

### Anaesthesia and Surgery

On anaesthesia days the guinea pigs were weighed 1 hour prior to anaesthesia and a subcutaneous injection of either meloxicam or saline given according to assigned group. Each individual was taken from the housing room to a theatre room using a rodent cage (RM3: 38x25x20cm North Kent plastic cages Ltd, Kent, UK). Anaesthesia was induced with sevoflurane in oxygen in an anaesthetic induction chamber (8% at 8L/min), and maintained using a face-mask (3–4% at 1.5 L/min). Guinea pigs were placed on a heat pad to maintain body temperature at 37–38°C (Harvard apparatus, Eldenbridge, Kent, UK) and eye lubricant applied (Carbomer 0.2% Eye Gel, Blumont Healthcare Ltd, Grantham, UK). The abdomen was clipped and sprayed with chlorhexidine surgical disinfectant (chlorhexidine gluconate 0.5%, Hydrex Pink, Ecolab Ltd., Leeds, UK). The duration of anaesthesia was standardised to 25 minutes to match the anaesthesia times for surgery. Following recovery from anaesthesia the guinea pigs were maintained in an incubator for 1 hour before being taken to the filming pen within the home room. Filming was conducted at the same time time of day as following surgery. After randomised allocation to treatment group, the order of treatment of each block of guinea pigs was retained for baseline, post-anaesthesia, post-surgery and 24h post-surgery, so that the time of each filming period, matched as closely as possible for each individual animal.

On surgery days, one hour prior to surgery, the guinea pigs were weighed and a subcutaneous injection of either meloxicam or saline given. Transport to the surgical theatre was as for the anaesthetic only day. Surgery began at 08:30 h with the same surgeon operating on all animals. The order of treatments was performed to a randomly allocated design to balance the effect of time of surgery. Anaesthesia was induced and maintained with sevoflurane and animals were prepared for surgery as described above. Guinea pigs underwent closed orchiectomy with 2 scrotal incisions approximately 2cm long (oriented on the long axis of the body) leaving the tunica vaginalis intact. The testicles were exteriorised, within the tunics, through the incision to expose the spermatic cord. The cord was crushed with haemostats and a ligature placed before transection and removal of the testis. Saline or the local anaesthetic mixture was infiltrated into the spermatic cord and tunics and a splash block applied to the subcutaneous tissues before the scrotal skin was closed with Vicyrl 4.0 (Johnson and Johnson, Belgium) with a subcuticular suture pattern. The surgical procedure was completed in 19 ± 2.8 minutes with a total duration of anaesthesia of 25±4.9 minutes. The guinea pigs were recovered within an incubator for 1 hour before being transferred to a filming pen. The testes were weighed after removal to allow adjustment of any post-surgery weight change.

### Video Recording

Guinea pigs were placed individually in a 90x60cm filming pen with two clear Perspex sides and 2 wire sides. The animals were filmed for 50 minutes using two High Definition video cameras (Sony Legria HFM506, Sony, Japan) placed at fixed distances from the two clear sides and one placed above the pen. No observer was present in the room at the time of filming. At baseline the guinea pigs were placed in the transport cage for 1 hour before being placed in the filming pen. This was done to match as closely as possible the sequence of events on the days when anaesthesia or surgery were carried out. On anaesthesia and surgery days the filming commenced 1h after the recovery from the anaesthetic. Baseline, 1h post-anaesthesia, 1h post-surgery and the Day post-surgery pre-meloxicam time-points were matched for time of day. 5h post-surgery and the Day post-surgery post-meloxicam time-points were matched for time of day.

### Behavioural scoring

The behaviour of the guinea pig was observed for 40 minutes of each recording and scored manually using Observe XT 11 (Noldus Information Technology, Netherlands) in a random order by a treatment and time-point blinded observer. Each file of video material was de-identified by removing the first few seconds of each segment that showed an identification card. The names of the video files were then changed to a random number that was generated in Excel (Microsoft) (one number per pair of videos i.e. video footage from the front and side). The videos were thus de-identified and watched according to this random number sequence in Observer so that the time-point, and guinea pig were presented randomly for viewing, with the observer blinded to treatment group and time point.

The ethogram used (Table 2) was developed based on previously recorded behaviours of rodents and rabbits following abdominal surgery, with additional behaviours added following preliminary viewing of material. Observer was used to generate frequency and/or duration of behavioural events (as appropriate) throughout the 40-minute observation period.

**Table 2:** Ethogram used for behavioural analysis.

Behaviour	Description
Abdominal contraction (frequency)	Contraction of the abdominal wall, often but not always associated with coprophagy
Bar chew (duration)	Chewing on the wire bars of the pen
Belly press (frequency)	Pressing of abdomen to cage floor
Chew (duration)	Chewing, often but not always immediately after eating (hay or food pellet no longer able to be seen at mouth—differentiates from eating)
Climb (duration)	Climbing the wire bars or the food hopper
Coprophagy (frequency)	Eating faecal pellets
Defaecate (frequency)	
Dig (duration)	
Drink (duration)	Drinking or interacting with the water bottle tube
Eat (duration)	Eating hay or pellets
Flinch (frequency)	Whole body contraction
Groom (duration)	Grooming body
Jump off hopper (frequency)	Returning to floor from food hopper
Lay down (frequency and duration)	Recumbent—legs under body
Lay down hind leg extension (frequency and duration)	Recumbent—one leg in extended to behind the body
lay down hind leg to side (frequency and duration)	Recumbent—one hind leg extended to the side of the body
Rear (frequency)	Standing on hind legs erect
Rear leg lift (frequency)	Momentary lifting of rear paw
Run (duration)	
Scratch (frequency)	
Shake (frequency)	
Sit on hopper (frequency and duration)	Positioned on top of the food hopper
Sleep (duration)	Presumptive sleep- eyes closed, no body movement
Stand (duration)	
Twitch (frequency)	Very rapid contraction of back muscles
Urinate (frequency)	
Vocalise (frequency)	
Walk (duration)	
Writhe (frequency)	Slow contortion of abdominal flank muscles
Yawn (frequency)	
Yawn/ stretch (frequency)	Stretch and yawn simultaneously

### Activity measurement

Video tracking software (ANY–maze version 4.98, San Diego Instruments, USA) was used to determine the distance travelled and time spent immobile in a 40-minute sequence. The immobility sensitivity was 50% with a minimum immobility period of 30 seconds.

Since there was an obvious preference for the guinea pigs to spend time in the back corner furthest from the transparent sides of the filming pen and next to the food hopper and water bottle. This corner was defined in ANY-maze to evaluate the automated behaviour measures related to this position in the pen.

### Body weight

Each guinea pig was weighed one hour prior to the anaesthesia and surgical procedure and then 24 and 48 hours post-procedure.

### Composite Scores (Phase 1 only)

Composite scores of several behavioural patterns were calculated in order to deal with the low frequency and duration of behaviours that were considered as potential indicators of post-surgical pain based on previous studies in rodents and rabbits [4,9,19]. It was hoped that these composite scores would be candidates for differentiating between treatment groups. The frequencies of lying down with an abnormal hind leg position (either to the side of the body or extended behind the body), hind leg lift, writhe and flinch were summed to give a composite ‘pain’ score. The durations of walk/ run, chewing bars, climbing, sitting on hopper, drinking and rearing summed to form a composite ‘active behaviour’ score.

### Statistical Methods

All statistical analyses were conducted using GraphPad Prism version 6.00 for Windows, (GraphPad Software, La Jolla California USA). Parametric analysis was carried out as this is a routinely used method to assess two factor designs. ANOVA is considered sufficiently robust to deal with this type data even if the data violates the normality assumption [20, 21, 22]. We considered the potential loss of power acceptable as this is a pilot study. Two-way repeated measures ANOVA was chosen as it allows us to compare two factors; treatment (between-subjects) and time points (within-subjects). Post-hoc analysis of treatment group effects were conducted using Sidak's multiple comparisons test. The Sidak post-hoc test was chosen as it is considered a powerful method when selecting a specific set of means for comparison.

In order to analyse the bodyweight data, actual body weights were compared between the groups prior to anaesthesia alone and anaesthesia and surgery. Further, the change in weight between one hour prior to the anaesthesia and surgical procedure and 24 and 48 hours post-procedure were calculated. This data was then compared between the treatment groups using unpaired t-tests.

## Results

### Phase 1 (placebo controlled phase)

The durations of the manually scored behaviours for all time periods are presented in Table 3. As the pattern for eating and chewing were similar (and it was sometimes difficult to delineate between the two behaviours from the video footage) the durations were combined before analysis. Results of the analysis over all time points in both phases of the study are presented below.

**Table 3:** Frequency or duration of individual behaviours in the control (no analgesia) and treated (meloxicam and local anaesthesia) groups. Values are mean +/- SEM.

	Phase 1										Phase 2			
	Baseline		Anaesthesia 1 hr		Anaesthesia 5 hr		Surgery 1 hr		Surgery 5 hr		Day post-surgery (pre meloxicam)		Day post-surgery (post meloxicam)	
	Control	Treated	Control	Treated	Control	Treated	Control	Treated	Control	Treated	Control	Treated	Control	Treated
Abdominal contraction (frequency)	5.3 ±1.7	5.6 ±1.7	10.9± 3.1	5.3 ± 0.9	7.4± 1.8	7.4± 3.7	19.6± 3.9	10.8±1.0	42.0± 0.1	11.5± 2.5	15.5± 4.0	8.1± 1.7	20.8± 6.3	6.5± 1.9
Bar chew (duration)	333.9± 170.2	85.4± 47.3	66.5± 38.1	22.1± 13.6	127.1± 84.9	163.6± 124.7	1.4± 0.9	41.7± 19.8	0.0± 0.0	59.0± 48.7	8.3± 6.1	238.4± 94.1	29.5± 29.5	217.0± 102.9
Belly press (frequency)	0.3± 0.3	0.0±0.0	0.0± 0.0	0.1± 0.1	0.3± 0.2	0.1± 0.1	0.1± 0.1	0.0± 0.0	0.0± 0.0	0.1± 0.1	0.0± 0.0	0.5± 0.3	0.0± 0.0	0.3± 0.3
Chew (duration)	483.5± 120.4	723.7± 182.0	986.6± 178.8	611.8± 124.3	686.6± 166.4	595.6± 146.9	902.0± 121.9	862.3± 111.0	37.4± 13.5	1014.1± 142.3	746.5± 227.4	732.3± 145.6	583.0± 149.2	956.7± 174.2
Climb (duration)	10.2± 3.8	6.2± 3.2	2.9± 2.9	0.9± 0.9	0.3± 0.3	1.1± 1.1	0.0± 0.0	0.7± 0.5	0.0± 0.0	0.0± 0.0	1.7± 1.7	1.1±0.6	0.0± 0.0	0.9± 0.9
Coprophagy (frequency)	8.0± 3.0	11.6± 5.5	6.0± 1.8	7.0± 2.2	2.8± 1.3	10.1± 3.8	12.8± 4.5	2.3± 1.0	7.3± 2.9	4.0± 2.0	9.5± 2.2	7.3± 2.3	5.5± 3.5	7.4± 4.4
Defeacate (frequency)	0.3± 0.2	0.5± 0.2	0.0± 0.0	0.3± 0.3	0.5± 0.3	1.4± 0.9	0.1± 0.1	2.3± 1.4	0.5± 0.5	0.0± 0.0	0.6± 0.4	1.4± 0.8	0.0± 0.0	0.8± 0.5
Dig (frequency)	1.5± 1.1	1.5± 1.1	0.6± 0.3	0.0 ±0.0	0.0 ± 0.0	0.3± 0.3	0.1 ± 0.1	0.1± 0.1	0.0± 0.0	0.3± 0.3	0.4± 0.3	0.0± 0.0	0.6± 0.6	0.3± 0.2
Drink/play with drinker (duration)	32.3± 8.6	57.3± 25.6	118.4± 69.5	93.2± 53.2	99.5± 43.3	25.3± 18.2	7.7± 4.0	28.4± 12.5	0.0± 0.0	65.1± 35.6	17.3± 16.8	20.6± 7.6	7.8± 3.6	34.2± 22.0
Eat (duration)	86.4± 22.1	120.9± 28.6	124.3± 26.2	110.3± 33.4	96.3± 35.2	223.7± 121.0	377.8± 96.0	294.1± 60.3	0.0± 0.0	294.3± 79.4	192.8± 93.7	282.1± 65.8	139.2± 62.8	347.4± 134.7
Flinch (number)	0.0± 0.0	0.0± 0.0	0.0± 0.0	0.0± 0.0	0.0± 0.0	0.0± 0.0	0.13± 0.13	0.0± 0.0	0.0± 0.0	0.0± 0.0	0.0± 0.0	0.0± 0.0	0.0± 0.0	0.0± 0.0
Groom (duration)	12.8± 6.0	18.1± 6.5	49.7± 21.6	59.7± 28.2	14.1± 5.4	12.9± 6.4	97.1± 45.0	61.3± 17.4	0.0± 0.0	39.0± 10.2	20.4± 8.1	32.2± 9.4	32.0± 26.3	44.0± 18.1
Jump off hopper (frequency)	5.5± 2.0	1.6± 1.2	3.1± 3.1	0.4± 0.4	0.3± 0.3	0.8± 0.7	0.0± 0.0	1.0± 0.7	0.0± 0.0	0.0± 0.0	0.3± 0.2	0.9± 0.7	0.0± 0.0	1.0±1.0
Lay down (frequency)	0.0± 0.0	0.0± 0.0	0.1± 0.1	0.1± 0.1	0.1± 0.1	0.0± 0.0	1.1± 0.5	0.0± 0.0	0.0± 0.0	0.8± 0.5	0.3± 0.2	0.0± 0.0	0.5± 0.3	0.0 ±0.0
Lay down (duration)	0.0± 0.0	0.0± 0.0	10.9± 10.9	8.1± 8.1	61.8± 61.8	0.0± 0.0	185.5± 89.6	0.0± 0.0	0.0± 0.0	243.0± 142.2	47.6± 31.4	0.0± 0.0	225.9± 202.9	0.0± 0.0
Lay down hind leg extended or to side (frequency)	0.0± 0.0	0.0± 0.0	0.0± 0.0	0.0± 0.0	0.0± 0.0	0.0±0.0	1.9± 0.6	0.0± 0.0	0.0± 0.0	0.1± 0.1	0.0± 0.0	0.0± 0.0	0.0± 0.0	0.0± 0.0
Lay down hind leg extended or to side (duration)	0.0± 0.0	0.0± 0.0	0.0± 0.0	0.0±0.0	0.0± 0.0	17.2± 17.2	36.2± 25.9	0.0± 0.0	0.0±0.0	16.6± 16.6	0.0± 0.0	0.0± 0.0	0.0± 0.0	0.0± 0.0
Rear (frequency)	25.0± 4.8	25.5± 6.8	6.4± 4.2	4.9± 2.0	6.9± 2.2	6.4± 1.8	0.6± 0.5	5.4± 2.5	0.0± 0.0	0.9± 0.6	2.5± 1.2	4.9± 1.7	0.8± 0.4	2.3± 1.2
Rear leg lift (frequency)	0.0± 0.0	0.0± 0.0	0.0± 0.0	0.0± 0.0	0.0± 0.0	0.0± 0.0	2.1± 1.4	0.0± 0.0	0.3± 0.2	0.0±0.0	0.0± 0.0	0.0± 0.0	0.0± 0.0	0.1± 0.1
Run/ walk duration (duration)	197.21± 18.51	288.32± 56.93	79.62± 28.16	102.38± 34.83	88.97± 39.97	101.03± 38.85	53.01± 21.32	148.43± 47.30	0.00± 0.00	42.25± 28.86	82.74± 60.49	146.19± 51.14	6.88± 4.60	52.24± 18.98
Scratch (frequency)	0.9± 0.4	0.3± 0.2	1.8± 0.5	2.3± 0.8	0.9± 0.5	0.3± 0.2	1.4± 0.8	1.4± 0.4	0.0± 0.0	0.9± 0.5	0.3±0.3	0.8± 0.3	0.0± 0.0	0.9± 0.2
Shake (frequency)	2.3± 0.5	2.1± 1.0	2.4± 0.4	2.3± 0.7	1.6± 0.3	0.8± 0.3	2.0± 0.7	2.5± 1.0	0.0± 0.0	1.0± 0.4	1.0± 0.4	2.4± 0.4	0.6± 0.2	1.5± 0.4
Sit on hopper (frequency)	4.4± 2.1	3.0± 1.6	3.5± 3.5	0.5± 0.5	0.3± 0.3	0.8± 0.7	0.0± 0.0	0.9± 0.6	0.0± 0.0	0.0± 0.0	0.0± 0.0	1.0± 0.7	0.0± 0.0	0.9± 0.9
Sit on hopper (duration)	156.84± 129.7	68.57± 39.0	192.91± 192.9	19.23± 19.2	47.81± 47.8	24.57± 24.5	0.0± 0.0	9.36± 7.1	0.0± 0.0	0.0± 0.0	0.0± 0.0	17.92± 14.6	0.0± 0.0	17.40± 7.4
Sleep (duration)	0.0±0.0	0.0± 0.0	0.0± 0.0	0.0± 0.0	0.0±0.0	0.0± 0.0	0.0± 0.0	0.0± 0.0	0.0±0.0	0.0± 0.0	0.0± 0.0	0.0± 0.0	13.1± 13.1	0.0±0.0
Stand (duration)	1766.6± 132.6	1763.1± 158.4	2029.8± 229.2	2083.8± 72.4	2015.7± 99.2	2047.4± 130.5	1888.1± 145.3	2132.3± 87.7	2400.2± 0.0	2040.1± 146.8	2172.3± 85.2	2126.6± 71.5	2112.6± 197.0	2244.0± 49.1
Urinate (frequency)	0.13± 0.13	0.38 ±0.18	0.13± 0.13	0.00± 0.00	0.13± 0.13	0.38± 0.38	0.13± 0.13	0.13± 0.13	0.00± 0.00	0.13± 0.13	0.00± 0.00	0.25± 0.16	0.00± 0.00	0.13± 0.13
Vocalise (frequency)	0.0± 0.0	0.4± 0.3	0.0± 0.0	0.0± 0.0	0.0 ± 0.0	0.0± 0.0	0.0± 0.0	0.0± 0.0	0.0± 0.0	0.3± 0.3	0.0± 0.0	0.0± 0.0	0.0± 0.0	0.0± 0.0
Writhe (frequency)	0.0±0.0	0.0± 0.0	0.0± 0.0	0.0± 0.0	0.0±0.0	0.0± 0.0	1.9± 1.6	0.0±0.0	0.0± 0.0	0.0± 0.0	0.0± 0.0	0.0±0.0	0.0± 0.0	0.0± 0.0
Yawn (frequency)	0.1± 0.1	0.0± 0.0	0.9±0.9	0.9± 0.5	1.6± 1.2	0.9± 0.6	0.4± 0.4	0.5± 0.4	0.1±0.1	2.0± 0.8	0.0± 0.0	0.0± 0.0	0.6± 0.4	0.0± 0.0
Yawn/ stretch (frequency)	0.13± 0.13	0.0± 0.0	0.0± 0.0	0.0± 0.0	0.0±0.0	0.0± 0.0	0.0± 0.0	0.0± 0.0	0.0±0.0	0.13± 0.13	0.0± 0.0	0.0±0.0	0.0± 0.0	0.0± 0.0

Individual behaviours showed few significant differences between the treatment groups. Following a significant ANOVA result (P = 0.03), Sidak’s multiple comparison test showed that guinea pigs in the control group showed the hind-leg lift behaviour significantly more than analgesic treated guinea pigs 1 hour after surgery (P = 0.0007). Following a significant ANOVA result (P = 0.007), Sidak’s multiple comparison test also showed that the control group displayed the behaviour of lying with the hind leg extended to the side or behind the body for a significantly longer duration than the analgesic treated group 1 hour post-surgery (P = 0.0003). Five out of eight control guinea pigs displayed this behaviour 1 hour post-surgery compared to no guinea pigs from the analgesic treated group.

**Fig 1:**
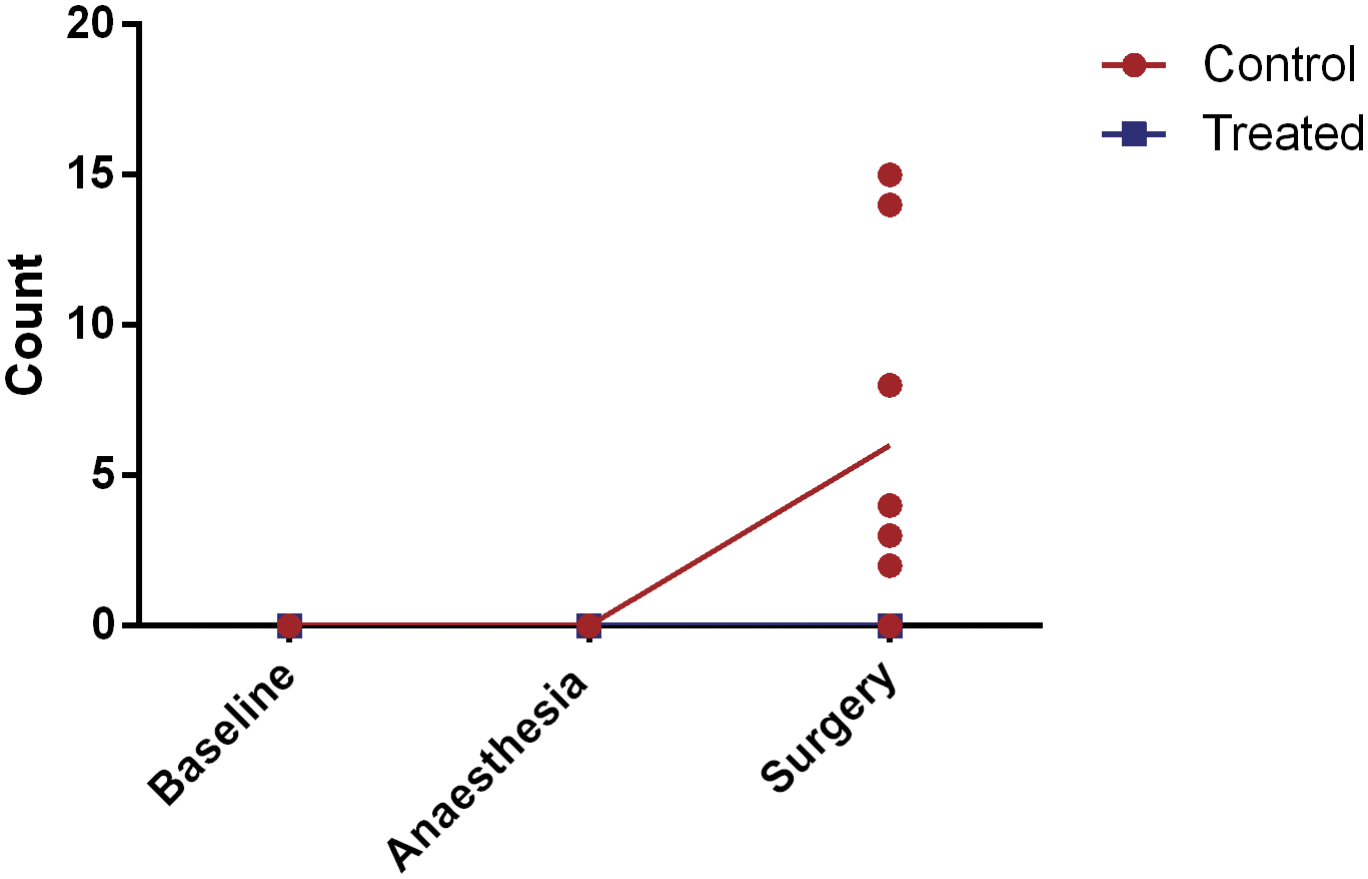
Composite pain score at baseline, after anaesthesia and 1 hour post-surgery. Values are individual data points and means.

For the composite pain score (abnormal hind leg position, hind leg lift, writhe and flinch), all but one guinea pig from the control group showed at least one abnormal behaviour at the 1 hour post-surgery recording (P<0.0001, Fig 1). This composite pain score at baseline, and 1h post-anaesthesia alone differed significantly from the score at 1h post-surgery in the control (no-analgesia) guinea pigs (P<0.0001) but not in the analgesic treated animals (P>0.1).

**Fig 2:**
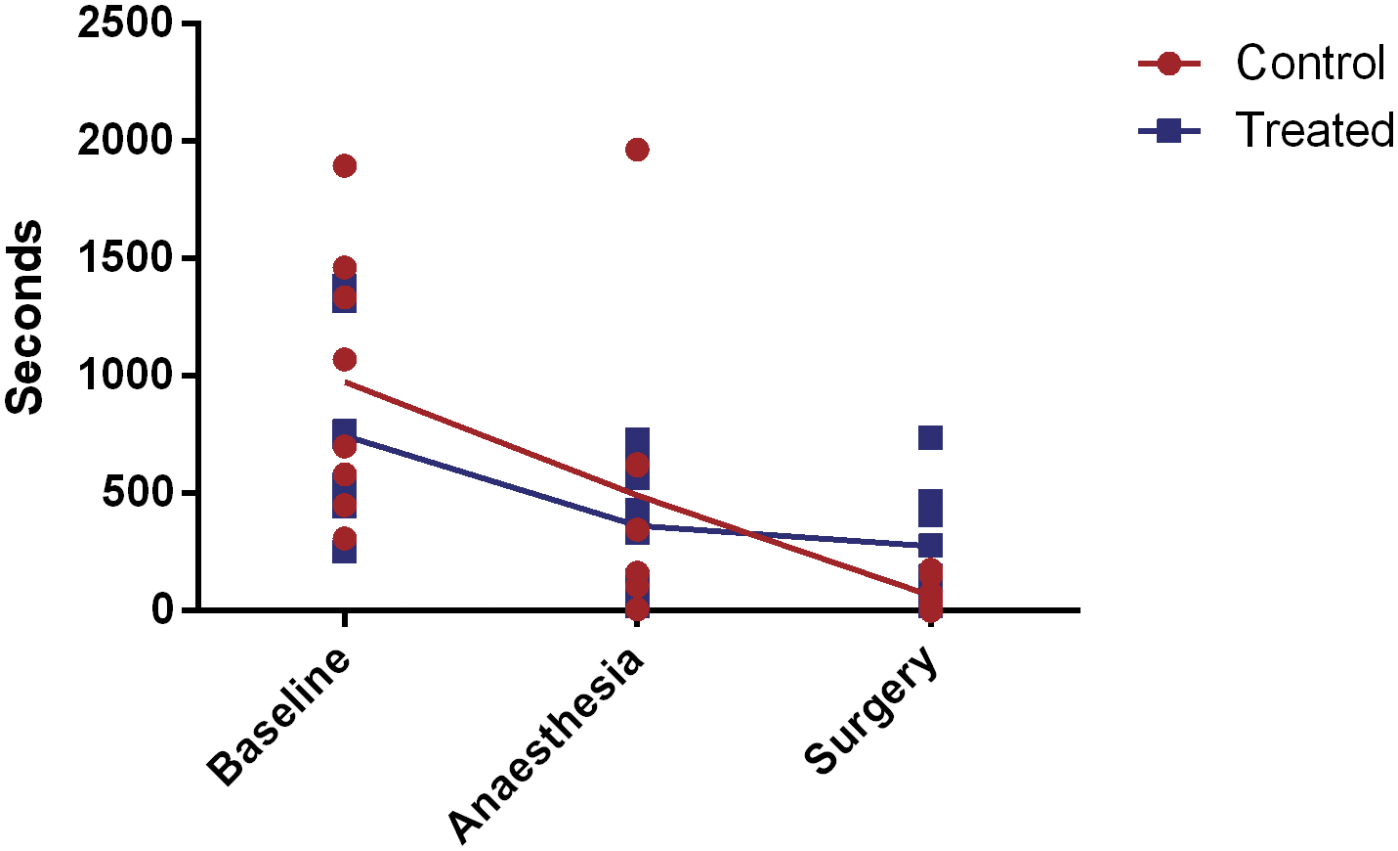
Composite active behaviour score at baseline, after anaesthesia and 1 hour post-surgery hours post-surgery. Values are individual data points and means.

The composite active behaviour score (walk/ run, chewing bars, climbing, sitting on hopper, drinking and rearing) showed some individual effects over time but did not differentiate treatment effects. The composite active behaviour score differed significantly over time (P<0.0001) but the time spent performing these behaviours at 1 hour post-surgery only differed from baseline (P<0.0001) and not from any other time points including anaesthesia (Fig 2). The composite active behaviour score similarly showed no difference between treatment groups overall (P = 0.3557) or at one hour post-surgery (P = 0.8550).

### Phase 2 (Day post-surgery, pre and post meloxicam administration)

#### Manual behaviour analysis

Details of all behaviours, at all time points, are given in Table 3. There was a significant effect of time (P = 0.0362) and a significant interaction between time and treatment group (P = 0.0003) on eating/drinking duration. For the control group there was a sharp decline in eating and chewing at the 5 hour post-operative time point (after administration of meloxicam). Sidak's multiple comparisons showed that this was the only time point where the there was a significant difference between treatment groups, with the control group spending significantly less time eating and chewing than the treated group (P<0.0001). While there was a difference in the time spent drinking/playing with drinker over time (P = 0.0315), there was no difference between groups for this behaviour.

There was a significant effect of time on the number of abdominal contractions (P<0.0001) and a significant interaction between time and treatment group (P = 0.0015). Sidak's multiple comparisons test showed that the control group performed significantly more abdominal contractions at the 5 hour post-operative time point point (after administration of buprenorphine) than the treated group (P<0.0001). There was no significance difference for the number of coprophagic events over time or between treatment group.

There was a significant effect of time on the duration spent chewing the bars (P = 0.0445), grooming (P = 0.0042) and the number of yawns (P = 0.0335), with baseline being significantly higher than 1h and 5h post-surgery (P = 0.0314, P = 0.0441).There was no significant effect of treatment group for any of these behaviours.

There was a significant effect of time on the duration of time spent walking or running (P<0.0001). There was evidence that the treated group spent more time walking or running (P = 0.0354) but this difference was not significant at any individual time point. There was a significant effect of time on standing (P = 0.0145) and behavioural shake (P = 0.0028), with baseline being significantly higher than both 1h and 5h post-surgery. There was no significant difference for these behaviours between the treatment groups.

There was a significant effect of time on the behaviours of rearing (P<0.0001), climbing (P<0.0001), and sitting on top of the food hopper (P = 0.0130), with baseline being significantly higher than the other time-points, except 1h post-anaesthesia. There was no significant difference between the treatment groups at any time points.

Belly press was a very infrequently observed behaviour and there was no significant difference over time or between treatment groups at any time. Writhing was also a very infrequently performed behaviour. It only occurred at 1 hour post-surgery in two control guinea pigs. Hind leg lift was also infrequently performed. There was a significant difference in the occurrence with time (P = 0.0447) and a significant interaction (P = 0.0365). As stated above, Sidak’s multiple comparison test showed that guinea pigs in the control group showed the behaviour significantly more than analgesic treated guinea pigs 1 hour after surgery (P = 0.0007).

There were no significant differences in the total duration of lying down or lying down with legs positioned underneath the body either over time or between treatment groups. There was, however an interaction between time and treatment group for both total duration of lying down and the duration of lying down with legs positioned underneath the body. The duration of time spent lying down with a hind leg positioned to the side or extended backwards showed a significant effect of time (P = 0.0209), a significant interaction between time and treatment group (P = 0.0070). As stated above, Sidak’s multiple comparison test showed that the control group displayed this behaviour for a significantly longer duration than the analgesic treated group 1 hour post-surgery (P = 0.0003). Five out of eight control guinea pigs displayed this behaviour 1 hour post-surgery compared to zero guinea pigs from the analgesic treated group. 5 hours post-surgery (after administration of buprenorphine to the control group) only one guinea pig (from the treated group) displayed this behaviour.

At 1 hour post-surgery the mean latency until any form of lying down was observed was 16 minutes with a range in the 5 animals of between 2 to 36 minutes. At 5 hours post- surgery the mean time was 24.5 minutes with a range in the 3 animals of 13 to 30 minutes.

Vocalisation was extremely rare with only 3 guinea pigs (2 at baseline and 1 at the 5 hour post-surgery recording) vocalising at any of the behaviour recordings.

#### Automated behaviour scoring

There were missing data points at the baseline for two guinea pigs in the treated group due to a malfunction in the video recording. The mean value at baseline of the other 14 guinea pigs was used in the analysis as a substitute for these missing data points. None of the automated measures differentiated between control and treated animals in phase 1 of the study.

**Fig 3:**
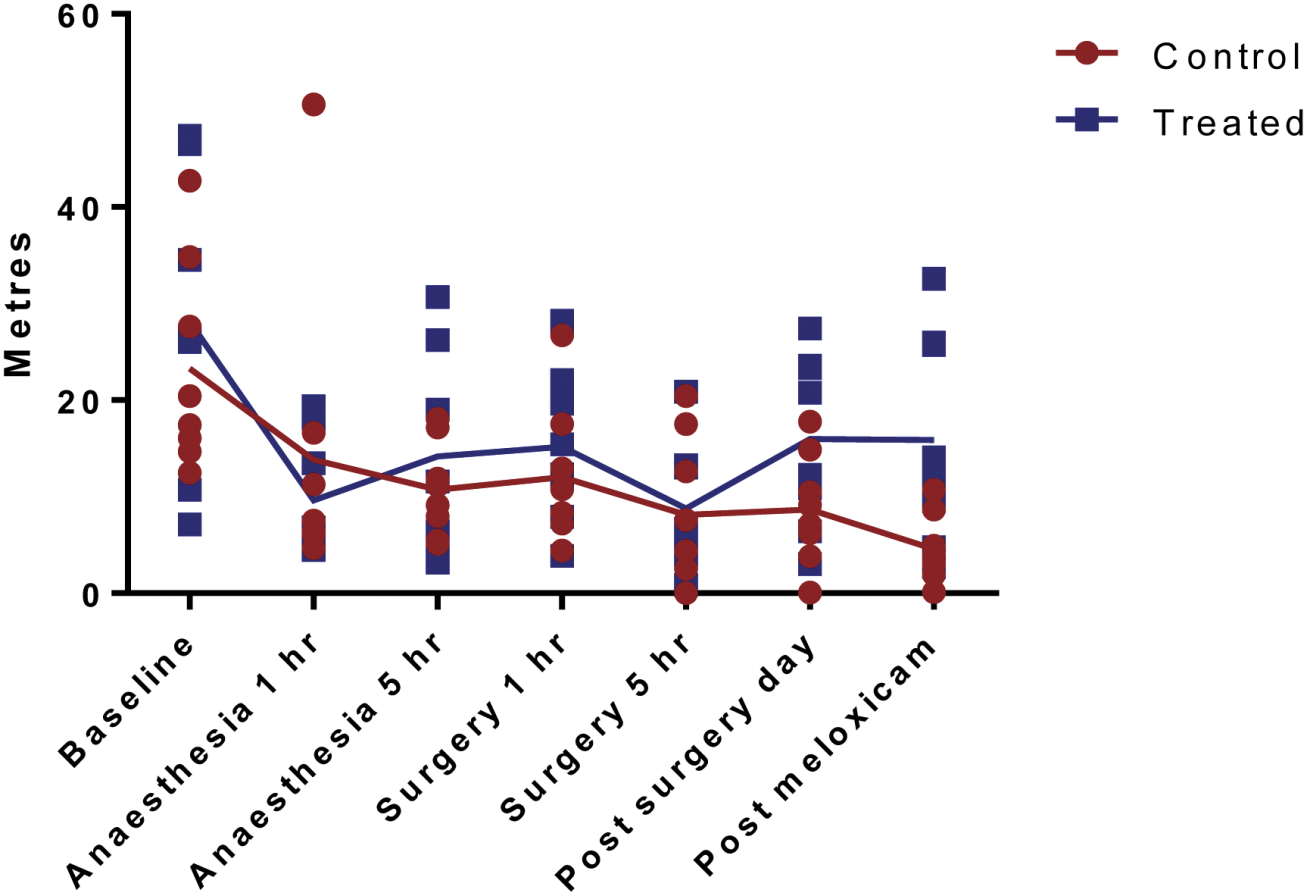
Distance Travelled, measured using automated behaviour score. Values are individual data points and means.

**Fig 4:**
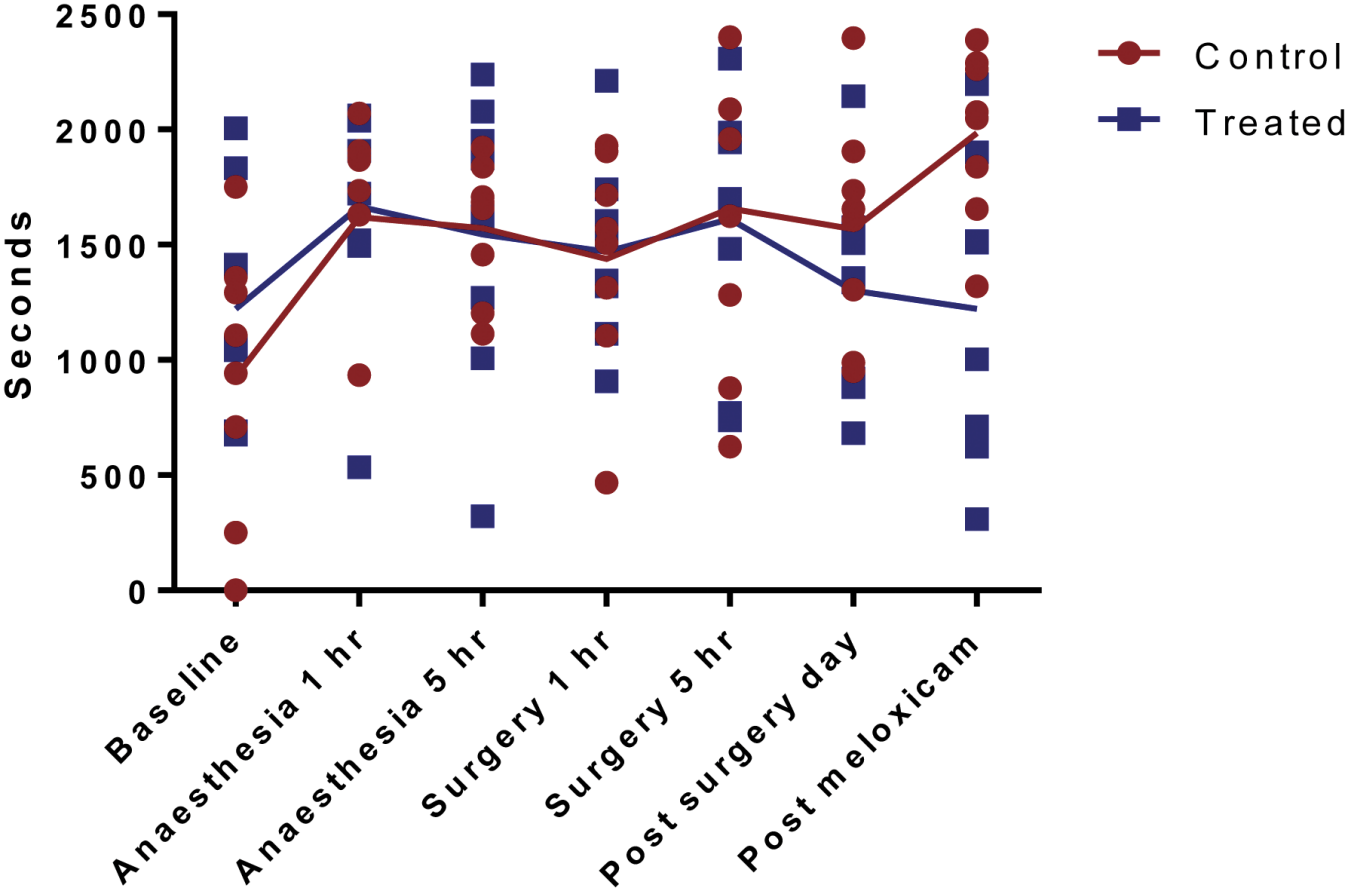
Time spent immobile, measured using automated behaviour score. Values are individual data points and means.

**Fig 5:**
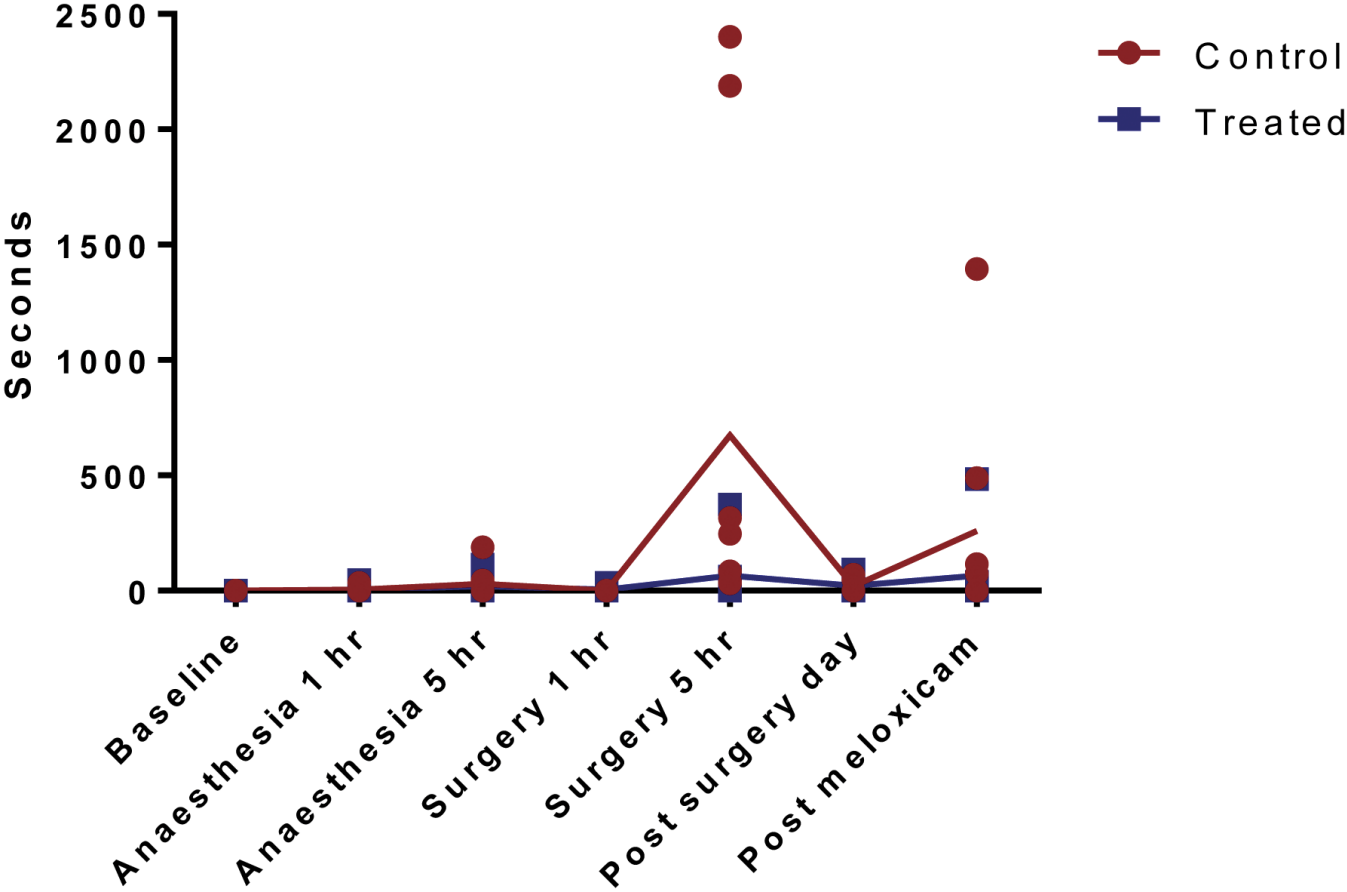
Latency to mobility, measured using automated behaviour score. Values are individual data points and means.

For Phase 2, the distance travelled was significantly affected by time (P<0.0001)(Fig 3), with baseline being significantly higher than the other time-points. For time immobile there was also a significant effect of time (P = 0.0189) with a significant interaction between time and treatment group (P = 0.0905). Sidak’s multiple comparison test showed that after the administration of meloxicam at the 1 day post-surgery time-point the control group spent significantly more time immobile (P = 0.0276)(Fig 4). For the latency until mobility there was a significant effect of time (P = 0.0063) and an interaction between time and treatment group (P = 0.0473)(Fig 5). Sidak’s multiple comparison test showed that at 5 hours post-surgery (after administration of buprenorphine), the control group had a significantly longer time until first mobility compared to the treated group (P = 0.0009). The time until first mobility was significantly longer than at 5 hours post-surgery compared to 5 hours post-anaesthesia (P = 0.0025).

For the time spent in the corner and time spent immobile in the corner there were no significant effects of time or treatment group. Latency to leave the corner appeared to increase over time but since not all guinea pigs started the behaviour recording periods in the corner no formal analysis was made.

#### Body weight

Before the anaesthetic there was no significant difference in the body weight of the guinea pigs in the different groups ((t(14) = 1.067, P = 0.3038), and no significant difference between groups prior to anaesthesia and surgery ((t(14) = 1.384, P = 0.1880). For the change in bodyweight from before to after general anaesthesia alone there was no significant difference between groups (t(14) = 0.3698, P = 0.7170). By 24h Post-surgery, the control group showed a greater loss in bodyweight (t(14) = 2.267, P = 0.0397). At 48 hours post-surgery there was no significant difference in bodyweight change between the groups.

## Discussion

The aim of this study was to identify behaviours that could potentially be developed into an objective, behaviour-based pain scoring system in guinea pigs undergoing surgery, as has been developed for other species. The surgical procedure (orchiectomy) was selected as it was expected to produce both somatic pain from the transection of skin and subcutaneous tissues as well as visceral pain from the transection of the spermatic cord and tunica vaginalis.

Meloxicam was administered prior to surgery to ensure the drug would be active during both the surgical procedure and in the post-operative period. In the veterinary clinical literature four NSAIDs are most frequently suggested for use in the guinea pig: Carprofen, Flunixin, Meloxicam and Metamizol (dipyrone) [23]. Meloxicam was chosen in this study to reflect its common usage in veterinary practice [24]. The dose was chosen based on current clinical recommendations. Suggested dosages from the literature for meloxicam in guinea pigs range from 0.1-≥0.5 mg/kg sc or po q24h [23,24, 25, 26,27] A dose of 0.2mg/kg sc was selected for this study based on this range of recommendations. Since the primary effects of NSAIDs on post-surgical pain are a reduction of hyperalgesia, local anaesthetic block with a combination of lidocaine and bupivacaine was also used to provide immediate and longer-term sensory nerve block.

The dose recommendations in the veterinary clinical literature are all based on clinical experience, since there are no previous published studies using pain-assessment schemes to evaluate analgesic dose rates in guinea pigs. In other small mammals, results from studies using different nociceptive stimuli have been used to establish likely effective dose rates of analgesics [28]. There are relatively few assessments of analgesics in guinea pigs using nociceptive stimuli. Intradermal injection to the back/flank of substances such as bradykinin resulted in biting, licking and scratching of the affected area; vigorous shaking of the head and body; backing, kicking, circling, rearing on the hind legs, biting the cage and squealing [29]. Application of an artery clip to the base of a toe elicits an audible squeak response that can be suppressed by analgesic agents [30]. Electric shocks have also been associated with vocalisation [31, 32]. However, all reports of anti-nociceptive testing appears to have been restricted to acute withdrawal methods, which have limited relevance to post-surgical pain control [33].

No observer was present in the room at the time of filming. The effect of an observer in the room has been noted to be especially important in some prey species such as the rabbit and may have been associated with early study failures to determine pain related behaviours in the rabbit [19]. Despite our best efforts to reduce potentially disruptive stimuli, there were still extraneous noises from other activities being conducted in the building. These however were random and with the random allocation of surgery order, the effects, if any, should not have biased the difference between the two groups of animals. Although behavioural changes which are potential indicators of pain were detected, it is important to note that observation of post-surgical animals may not always be under the conditions employed in this study, especially in busy veterinary practices or research facilities. The development of practical scoring systems for such settings must be robust to such potentially noisy conditions.

Rabbits experiencing stress or experiencing pain or discomfort may respond with immobility when placed in unfamiliar environments, [34]. Guinea pigs have been considered to respond similarly, and on average the guinea pigs in this study spent a large proportion of their time immobile. The most active time point was the baseline behaviour recording period. It is likely that part of the reduction in activity was related to habituation to the environment and reduction in escape behaviours. There was generally low activity of guinea pigs after surgery and the difference between groups at the 1 hour post-surgery recording was considered the most important in terms of identifying specific pain-related behaviours.

Individual abnormal (pain-related) behaviours were observed very infrequently, hence a composite score is considered potentially most useful as a means of assessing pain in this species. Of the individual abnormal behaviours the most potentially useful behaviour was a change in posture. Guinea pigs were seen to lay down with the hind legs tucked under the body, sometimes accompanied by abnormal positioning of a hind leg either positioned to the side or extended behind the body. This posture was the most obvious early (phase 1) post-surgery difference between analgesic and control animals. Although being the most consistent pain-related behaviour it had a variable, and in some cases long latency.

Active behaviours were also considered in this study as a potential way to recognise the difference between treatment groups and so identify indicators that might predict the absence of pain. At the 1 hour post-surgery recording (phase 1), individual manually scored active behaviours did not clearly distinguish between the guinea pigs that had and had not received analgesia. There was also no statistically significant trend for guinea pigs that had received analgesia to better maintain their overall performance (composite score) of the active behaviours walk/run, rear, climb, sit on top of the hopper, chew bars and drink/ play with drinker. These results indicate that it may be difficult to develop active behaviours into a useful post-surgery pain assessment scheme based on the results of this study.

Manual behaviour scoring is time consuming. Video footage was also processed by an automated behaviour tracking software (ANY-maze) to compare with the manual behaviour assessment of locomotor behaviour and determine if this less time-consuming analysis tool could differentiate between the treatment groups. It was found that none of the individual automated activity measures evaluated in the immediate post-surgical period distinguished between guinea pigs that did or did not receive analgesia. This finding is consistent with much of the literature in mice and rats using automated scoring of activity measures such as rearing and locomotion [35]. Differences between anaesthesia and surgery for the automated behaviour analysis were, for the most part, small and did not differentiate between the post-anaesthesia and post-surgery observation periods.

Recordings were continued into the later post-surgery period, but these results must be interpreted with care because of the administration of buprenorphine as a rescue analgesic in control groups after the first post-surgery behaviour recording. Use of intervention analgesia was a difficult ethical decision, since continued use of a placebo control group might have magnified the group differences at later time points. However, it was considered that in this exploratory study it should be possible to identify the major effects of surgery and analgesic treatment in the immediate post-operative period. This was confirmed by the differences in pain specific behaviours identified at the 1h time point (phase 1). The administration of the rescue analgesic was deemed necessary in the study design to alleviate potential ongoing pain and distress. We chose not to administer buprenorphine to the treated group so as to be able to follow this group’s behaviour for a longer period, and rescue analgesia was not considered necessary as it was assumed these animals would have sufficient pain relief. Published guidelines have suggested doses of buprenorphine ranging from 0.05–0.5 mg/kg [23,26] and a dose of 0.05mg/kg was selected for use in this study based on these suggested dose rates. Inclusion of a third group that comprised meloxicam and local anaesthetic, followed by administration of buprenorphine after the first post-surgery recording would have aided interpretation, but this would have also increased the number of animals used considerably and this was not considered appropriate in this preliminary study. Buprenorphine was selected for rescue analgesia as it has a more rapid onset than NSAIDs, and is a more potent analgesic. As stated in the materials and methods, intervention analgesia was also intended to be used in animals in the treated (analgesia) group if more than moderate signs of pain were noted, and it was considered an opioid would be likely to be more effective than a further dose of an NSAID. Other opioids could have been used, but there is very little data on their efficacy in guinea pigs. It is also likely that all opioids would have the same effects of modulating behaviour in both painful and pain free animals.

Using manual behaviour scoring, lying down behaviour was again noticed at the 5 hour post-surgery recording. At this time point, however, the behaviour was observed in the treated group and not the control group that had received rescue analgesia. No abnormal leg positioning accompanied the behaviour at this time. The later onset of this behaviour in the treated animals may indicate that these animals had experienced an early analgesic effect as a result of the local anaesthetic infiltration that was waning at the 5h time point.

At 5 hours post-surgery none of the control guinea pigs performed any active behaviours. If the buprenorphine administered after the 1 hour recording was having only an analgesic effect, the active behaviours would be expected to remain stable or increase, not decrease as was the case in this study. We suggest that a sedative effects of buprenorphine could be responsible for this effect.

Abdominal contractions appeared to increase after surgery especially in the control group. The difference between groups was, however, significant only at the 5 hour post-surgery time-point. This behaviour was seen at baseline, after anaesthesia and also the day after surgery. The behaviour was commonly associated with coprophagy and at least some the contractions could therefore be normal. A possible explanation for the increase after surgery is that the generically scored behaviour of abdominal contractions may have consisted of two different behaviours: contractions that were a normal part of the gastrointestinal function of guinea pigs and a second type of contraction that may be analogous to the writhing behaviour displayed in the rat or mouse after painful or noxious stimuli is applied to the abdominal area [4,7]. The difference between groups for this behaviour was significant at the 5 hour post-surgery time point. After the 1 hour post-surgery recording the control group had received buprenorphine by subcutaneous administration while the treated group received saline by the same route. It is possible that the increase in abdominal contractions for this group may be a non-specific effect of buprenorphine. Buprenorphine, in common with other opioids, can cause pica behaviour in rats [36, 37]. It may be that buprenorphine can have similar gastrointestinal effects in guinea pigs and this is manifested as increased abdominal contractions. The difference could also reflect the differences in analgesic regimens and in particular the use of pre-emptive analgesia compared to rescue analgesia.

Vocalisation has been associated with application of noxious stimuli such as electric shock in guinea pigs [31, 32] and is reported, more generally, as a sign of pain [17]. Vocalisation was not associated with surgery in this study and the frequency of vocalisations was 0–0.4 in all groups at all time points. These differences could be related to the type of stimulus. Application of pressure to the surgery site might have elicited vocalizations and could be a potential way to assess the effects of analgesic treatments. In other rodents species (mouse and rat) the use of vocalisation as a measure of pain has had mixed results [38].

Bodyweight is a simple measure that has been associated with beneficial effects of some analgesic agents. It is, however, not specific for pain as surgical stress can influence bodyweight changes greatly. Anaesthesia with or without meloxicam had minimal effects on bodyweight. This indicated meloxicam, at the administered dose, does not have significant non-specific effects on bodyweight. After surgery, the analgesic treated group (meloxicam and local anaesthetic) had less weight loss at 24 hours post-surgery than the control group. The difference between groups after surgery could indicate that the analgesic combination was effective at attenuating the effects of pain on bodyweight. It must, however, be considered that the control group received rescue analgesia using buprenorphine after the first post-operative behaviour recording. This addition might be expected to reduce the potential difference between the groups by minimising pain in the control group. However, buprenorphine in some rodents species (mouse and rat) has been associated with non-specific effects related to activity levels and food and water intake that could have subsequent effects on bodyweight [33] With the current study design, and the limited information available on the effects of buprenorphine in the guinea pig, it is therefore not possible to determine whether bodyweight was positively, negatively or unaffected by buprenorphine in the control group.

For this study guinea pigs were housed singly for the period of study and during filming. This was done in order to track the behaviour and activity of individual animals. Guinea pigs are a social species and isolation may have reduced the repertoire of potential behaviours. It would be interesting to study the effects of surgery under social housing conditions. Although this would increase the difficulty of scoring individual behaviour, changes in social behaviour that could be affected by pain and surgery might be detected. This has been shown to be of value in mice [39]. Social housing might also increase the general activity of the guinea pigs and make changes in overall activity levels easier to detect.

Overall there were few specific post-surgical indicators of pain identified in the current study. In addition, they were displayed infrequently. Similar conclusions were reached in a recent study of post-castration behaviour in guinea pigs [16)]. These authors noted that subtle body movements were seen following surgery (abdominal contractions, back arching, twitching and weight shifting), at a greater frequency than following anaesthesia alone. Their study did not assess changes to normal activity, but did note that time to consume a favoured food-stuff did not differ between groups. Their study did not evaluate the effects of analgesic treatment on these changes, but did establish that the abnormal behaviours occurred in parallel with changes in nociceptive thresholds (measured using an electronic Von Frey apparatus), and hence were likely to be pain related, rather than reflecting non-specific changes to surgery. The differences in abnormal behaviours noted in this study [16] and the present study may be related to differences in the behavioural sampling method used (interval sampling of 10 seconds for a total of 90 seconds, in comparison to 40 minutes continuous observation). Infrequent behaviours such as lying down with abnormal limb positioning might therefore not be noted sufficiently frequently. Although out study indicates that this behaviour (lying down, especially with abnormal hind leg positioning) could be a candidate indicator for studies aiming to determine analgesic efficacy after surgery in the abdominal region, we conclude that long periods of observation under very controlled conditions may be necessary. Since few specific behaviours were identified, and these occurred at relatively low frequencies, results from the present study indicate that clinical use of a behaviour-based pain scoring system may be of limited value in guinea pigs. In addition, it will be critical to determine if guinea pigs that are aware of being observed would display these behaviours at the same frequency or would suppress them. It will also be necessary to determine whether similar behaviours can be observed following other types of surgical procedure. At present, we would recommend that if any of these behaviours (lying down (especially with abnormal leg positioning), hind leg lift, writhe or flinch) are observed, the animal should be observed for longer, and additional analgesia administered.

Meloxicam appears to have little or no non-analgesic effects that would interfere with behaviour studies. In comparison the results from the control animals that received buprenorphine for rescue analgesia suggest that buprenorphine may have potential non-analgesic related effects. There was some indication that effects such as sedation could render the study of behaviour unreliable as an indicator of pain as a number of normal active behaviours were reduced after buprenorphine administration. Further research would be needed into the analgesic efficacy of buprenorphine to better interpret the results seen in this study. The effect of increasing abdominal contractions in association with the administration with buprenorphine is interesting and could indicate an opioid-induced adverse gastrointestinal effect that could be analogous to nausea, as has been speculated in other rodent species [40, 41]. Buprenorphine is a commonly used analgesic agent in both the laboratory and veterinary settings and it is important to understand what, if any, aversive effects commonly used drugs may have on the animals.

## Supporting Information

S1 Table Anymaze Data. (XLSX)

S2 Table Manually Scored Data. (XLSX)

## References

[1] Home Office (2014) Statistics of scientific procedures on living animals, Great Britain 2014, National Statistics, UK

[2] Pet Food Manufacturer's Association, Pet population 2015, available at http://www.pfma.org.uk/pet-population-2015

[3] Gebhart G. F., Basbaum A. I., Bird S. J., Flecknell P., Goodly L., Karas A. Z., S. T. et al (2009). Recognition and Alleviation of Pain in Laboratory Animals, The National Academies Press. 20662126

[4] Wright-Williams S. L., Courade J. P., Richardson C. A., Roughan J. V. and Flecknell P. A. (2007). "Effects of vasectomy surgery and meloxicam treatment on faecal corticosterone levels and behaviour in two strains of laboratory mouse." Pain 130(1): 108–118. 17196337 10.1016/j.pain.2006.11.003

[5] Langford D. J., Bailey A. L., Chanda M. L., Clarke S. E., Drummond T. E., Echols S., Glick S., Ingrao J., Klassen-Ross T. and LaCroix-Fralish M. L. (2010). "Coding of facial expressions of pain in the laboratory mouse." Nature methods 7(6): 447–449. 10.1038/nmeth.1455 20453868

[6] Leach M. C., Klaus K., Miller A. L., di Perrotolo M. S., Sotocinal S. G. and Flecknell P. A. (2012). "The Assessment of Post-Vasectomy Pain in Mice Using Behaviour and the Mouse Grimace Scale." PloS one 7(4): e35656 10.1371/journal.pone.0035656 22558191 PMC3338444

[7] Roughan J. V. and Flecknell P. A. (2001). "Behavioural effects of laparotomy and analgesic effects of ketoprofen and carprofen in rats." Pain 90(1): 65–74. 11166971 10.1016/s0304-3959(00)00387-0

[8] Roughan J. V. and Flecknell P. A. (2003). "Evaluation of a short duration behaviour‐based post‐operative pain scoring system in rats." European Journal of Pain 7(5): 397–406. 12935791 10.1016/S1090-3801(02)00140-4

[9] Roughan J. and Flecknell P. (2004). "Behaviour-based assessment of the duration of laparotomy-induced abdominal pain and the analgesic effects of carprofen and buprenorphine in rats." Behavioural pharmacology 15(7): 461–472. 15472568 10.1097/00008877-200411000-00002

[10] Sotocinal S. G., Sorge R. E., Zaloum A., Tuttle A. H., Martin L. J., Wieskopf J. S., Mapplebeck J. C. S., Wei P., Zhan S. and Zhang S. (2011). "The Rat Grimace Scale: A partially automated method for quantifying pain in the laboratory rat via facial expressions." Molecular pain 7(1): 55. 21801409 10.1186/1744-8069-7-55 PMC3163602

[11] Farnworth M., Walker J., Schweizer K., Chuang C., Guild S., Barrett C., Leach M. and Waran N. (2011). "Potential behavioural indicators of post-operative pain in male laboratory rabbits following abdominal surgery." Animal Welfare 20(2): 225–237.

[12] Keating S. C. J., Thomas A. A., Flecknell P. A. and Leach M. C. (2012). "Evaluation of EMLA Cream for Preventing Pain during Tattooing of Rabbits: Changes in Physiological, Behavioural and Facial Expression Responses." PloS one 7(9): e44437 10.1371/journal.pone.0044437 22970216 PMC3436883

[13] Liles J., Flecknell P., Roughan J. and Cruz-Madorran I. (1998). "Influence of oral buprenorphine, oral naltrexone or morphine on the effects of laparotomy in the rat." Laboratory Animals 32(2): 149–161. 9587897 10.1258/002367798780600025

[14] Arras M., Rettich A., Cinelli P., Kasermann H. and Burki K. (2007). "Assessment of post-laparotomy pain in laboratory mice by telemetric recording of heart rate and heart rate variability." BMC veterinary research 3(1): 16. 17683523 10.1186/1746-6148-3-16 PMC1965463

[15] Reinert J. (2012). Assessment of pain behavior of male guinea pigs after Castration—Effect of meloxicam Doctorate of Veterinary Medicine, Ludwig-Maximilians-University.

[16] Dunbar M.L. David E.M., Aline M.R., Lofgren J.L. (2016) Validation of a behavioural ethogram for assessing postoperative pain in the guinea pig. Journal of the American Association for Laboratroy Animal Science, 55, 29–34. PMC4747008 26817977

[17] Morton D. and Griffiths P. (1985). "Guidelines on the recognition of pain, distress and discomfort in experimental animals and an hypothesis for assessment." Veterinary Record 116(16): 431–436. 3923690 10.1136/vr.116.16.431

[18] Rehbinder C., Baneux P., Forbes D., Van Herck H., Nicklas W., Rugaya Z. and Winkler G. (1996). "FELASA recommendations for the health monitoring of mouse, rat, hamster, gerbil, guineapig and rabbit experimental units: Report of the Federation of European Laboratory Animal Science Associations (FELASA) Working Group on Animal Health accepted by the FELASA Board of Management, November 1995." Laboratory Animals 30(3): 193–208. 8843044 10.1258/002367796780684881

[19] Leach M. C., Allweiler S., Richardson C., Roughan J. V., Narbe R. and Flecknell P. A. (2009). "Behavioural effects of ovariohysterectomy and oral administration of meloxicam in laboratory housed rabbits." Research in Veterinary Science 87(2): 336–347. 10.1016/j.rvsc.2009.02.001 19303122

[20] Glass GV, Peckham PD, Sanders JR. Consequences of failure to meet assumptions underlying the fixed effects analyses of variance and covariance. Review of educational research. 1972 7 1;42(3):237–88.

[21] Harwell MR, Rubinstein EN, Hayes WS, Olds CC. Summarizing Monte Carlo results in methodological research: The one-and two-factor fixed effects ANOVA cases. Journal of Educational and Behavioral Statistics. 1992 12 21;17(4):315–39.

[22] Lix LM, Keselman JC, Keselman HJ. Consequences of assumption violations revisited: A quantitative review of alternatives to the one-way analysis of variance F test. Review of educational research. 1996 12 1;66(4):579–619.

[23] Kaiser S., Kruger C. and Sachser N. (2010). The Guinea Pig The UFAW Handbook on the Care and Management of Laboratory and Other Research Animals. Hubrecht R. C and Kirkwood J., Wiley**:** 380–398.

[24] Keown A., Farnworth M. and Adams N. (2011). "Attitudes towards perception and management of pain in rabbits and guinea pigs by a sample of veterinarians in New Zealand." New Zealand Veterinary Journal 59(6): 305–310. 10.1080/00480169.2011.609477 22040336

[25] Hawkins M. G. (2006). "The Use of Analgesics in Birds, Reptiles, and Small Exotic Mammals." Journal of Exotic Pet Medicine 15(3): 177–192.

[26] Flecknell P. (2015). Laboratory animal anaesthesia, Academic Press.

[27] Morrisey J. K. and Carpenter J. W. (2012). Ferrets, Rabbits and Rodents: Clinical Medicine and Surgery. St Louis, Elsevier Health Sciences.

[28] Liles J. H., & Flecknell P. A. (1992). The use of non-steroidal anti-inflammatory drugs for the relief of pain in laboratory rodents and rabbits. *Laboratory Animals*, 26(4), 241–255. 1447903 10.1258/002367792780745706

[29] Collier H. O. J. and Lee I. R. (1963). Nociceptive responses of guinea pigs to intradermal injections of bradykinin and kaludin-10. British Journal of Pharmacology and Chemotherapy 21(1): 155–164. 14066140 10.1111/j.1476-5381.1963.tb01511.x PMC1703871

[30] Collier H. O. J., Warner B. T. and Skerry R. (1961). Multiple toe pinch method for testing analgesic drugs. British Journal of Pharmacology and Chemotherapy 17(1): 28–40. 13694702 10.1111/j.1476-5381.1961.tb01101.x PMC1482071

[31] da Silva L. F. S. and Menescal-de-Oliveira L. (2006). "Cholinergic modulation of tonic immobility and nociception in the NRM of guinea pig." Physiology & Behavior 87(4): 821–827. 16545845 10.1016/j.physbeh.2006.01.019

[32] Favaroni Mendes L. A. and Menescal-de-Oliveira L. (2008). "Role of cholinergic, opioidergic and GABAergic neurotransmission of the dorsal hippocampus in the modulation of nociception in guinea pigs." Life Sciences 83(19–20): 644–650. 10.1016/j.lfs.2008.09.006 18854195

[33] Roughan J. V., & Flecknell P. A. (2002). Buprenorphine: a reappraisal of its antinociceptive effects and therapeutic use in alleviating post-operative pain in animals. *Laboratory Animals*, 36(3), 322–343. 12144743 10.1258/002367702320162423

[34] Turner P. V., Chen C. H. and Taylor M. W. (2006). "Pharmacokinetics of Meloxicam in Rabbits After Single and Repeat Oral Dosing." Comparative Medicine 56(1): 63–67. 16521861

[35] Miller A.L., Flecknell P.A., Leach M.C and Roughan J.V. (2011) A comparison of a manual and an automated behavioural analysis method for assessing post-operative pain in mice. Applied Animal Behaviour Science 131, 138–144

[36] Mitchell D., Krusemark M. L., & Hafner E. (1977). Pica: A species relevant behavioral assay of motion sickness in the rat. *Physiology & Behavior*, 18(1), 125–130. 561970 10.1016/0031-9384(77)90103-2

[37] Clark J. A., Myers P. H., Goelz M. F., Thigpen J. E., & Forsythe D. B. (1997). Pica behavior associated with buprenorphine administration in the rat. *Laboratory Animal Science*, 47(3), 300–303. 9241634

[38] Whittaker A. L. and Howarth G. S. (2013). "Use of spontaneous behaviour measures to assess pain in laboratory rats and mice: How are we progressing?" Applied Animal Behaviour Science(0).

[39] Jirkof P., Cesarovic N., Rettich A., Fleischmann T. and Arras M. (2012). "Individual housing of female mice: influence on postsurgical behaviour and recovery." Laboratory Animals 46(4): 325–334. 10.1258/la.2012.012027 23097566

[40] Aung H. H., Mehendale S. R., Xie J.-T., Moss J. and Yuan C.-S. (2004). "Methylnaltrexone prevents morphine-induced kaolin intake in the rat." Life sciences 74(22): 2685–2691. 15043984 10.1016/j.lfs.2003.08.047

[41] Thompson A. C., Kristal M. B., Sallaj A., Acheson A., Martin L. B. E. and Martin T. (2004). "Analgesic efficacy of orally administered buprenorphine in rats: methodologic considerations." Comparative medicine 54(3): 293–300. 15253276

